# Immunologically “cold” triple negative breast cancers engraft at a higher rate in patient derived xenografts

**DOI:** 10.1038/s41523-022-00476-0

**Published:** 2022-09-10

**Authors:** Varduhi Petrosyan, Lacey E. Dobrolecki, Emily L. LaPlante, Ramakrishnan Rajaram Srinivasan, Matthew H. Bailey, Alana L. Welm, Bryan E. Welm, Michael T. Lewis, Aleksandar Milosavljevic

**Affiliations:** 1 Department of Molecular and Human Genetics, Baylor College of Medicine, Houston, TX USA; 2 Lester and Sue Smith Breast Center, Baylor College of Medicine, Houston, TX USA; 3 Eccles Institute of Human Genetics, University of Utah, Salt Lake City, UT USA; 4 Huntsman Cancer Institute, University of Utah, Salt Lake City, UT USA; 5 Department of Oncological Sciences, University of Utah, Salt Lake City, UT USA; 6 Department of Surgery, University of Utah, Salt Lake City, UT USA; 7 Dan L Duncan Comprehensive Cancer Center, Baylor College of Medicine, Houston, TX USA; 8 Departments of Molecular and Cellular Biology and Radiology, Baylor College of Medicine, Houston, TX USA

**Keywords:** Breast cancer, Cancer microenvironment

## Abstract

TNBC is a heterogeneous subtype of breast cancer, and only a subset of TNBC can be established as PDXs. Here, we show that there is an engraftment bias toward TNBC with low levels of immune cell infiltration. Additionally, TNBC that failed to engraft show gene expression consistent with a cancer-promoting immunological state, leading us to hypothesize that the immunological state of the tumor and possibly the state of the immune system of the host may be essential for engraftment.

## Introduction

Experimentally tractable in vivo models are critical for understanding tumor biology, developing therapeutic interventions, and uncovering mechanisms of therapy resistance. Better understanding of model limitations is key to improving rigor and reproducibility of basic research. The success of clinical trials also critically depends on understanding biases of preclinical models. Historically, immortalized cell lines and genetically engineered mouse models have been used primarily to study breast cancer biology. However, in vitro models lack the tumor microenvironment^1^, and cell lines do not faithfully recapitulate the biology of the tumor of origin^2–4^. Previous studies have shown that as many as 20% of cell lines are cross-contaminated, and that the repeated passage of cell lines is associated with the accumulation of mutations not seen in the primary tumor^5^. Genetically Engineered Mouse Models (GEMMs) better represent the tumor microenvironment of primary tumors. However, because an individual GEMM yields a relatively homogeneous set of tumors, GEMMs do not model the full diversity of human breast cancer^6^. A possible exception is the mouse TP53-null mammary epithelial transplantation model. This model can generate tumors representing multiple tumor types, but the relationship between mouse TP53-null tumors and human tumors of various molecular subtypes remains unclear^7,8^.

To address some of the shortcomings of traditional cancer models, over the last two decades there has been significant progress in the development of Patient-Derived Xenografts (PDXs). Large PDX cohorts are now available for breast cancer, as well as other tumor types (https://pdxportal.research.bcm.edu/, https://pdmr.cancer.gov/, http://www.pdxnetwork.org, https://www.pdxfinder.org/). Breast cancer PDXs have been well characterized and shown to be biologically consistent with patient tumors across multiple “omics” types including mutations, copy number alterations, transcriptomics, and proteomics^6,9–15^. Despite advances in PDX modeling, not all breast cancers can be engrafted successfully as PDX models. Triple Negative Breast Cancer (TNBC) has a higher engraftment rate as PDXs (~60%) than hormone positive or HER2 + tumors (~10–15%)^11^, and are therefore the best represented breast cancer subtype in PDX collections. TNBCs lack the expression of the ESR1 (ER) and PGR (PR) steroid hormone receptors, and do not show amplification or overexpression of oncogenic ERBB2 (HER2). TNBCs are highly heterogenous, and multiple subtypes of TNBCs have previously been identified based on histopathology^16^, genomic alterations^17^, transcriptomic profiling^18^, a combination of mutational profiling and transcriptomic profiling^19^, and by the tumor microenvironment^20^.

Several TNBC subtyping methods have identified an immunomodulatory subtype, and TNBC has the highest proportion of immune cell-enriched “hot” tumors of all breast cancer subtypes^21^. A necessary disadvantage of PDX models is that an immunocompromised host (typically mouse) is needed to ensure that the patient tumor is not rejected by the immune system. The immune system is a double-edged sword that can promote tumor growth as well as target tumor cells for elimination^22^. As immunodeficient PDX models lack a functional immune system, we investigated whether the immune status of human breast cancers was related to growth as a PDX.

Because immunologically “hot” and “cold” tumors were shown previously to have distinct epithelial cancer cell profiles^23,24^, we asked if the cancer cell fraction in PDX models mostly matches the cancer cell profiles of the immunologically “hot” or “cold” tumors in patient tumors. Ideally, the state of cancer cells within PDXs should match the cancer cell state in human tumors. The cancer cell state in PDX models is readily ascertainable against the background of the mouse cells because the cancer cell expression profile can be separated from the bulk expression by determining which mRNA-sequencing reads have human origins^25^. To access the cancer cell state in human primary tumors from readily available bulk mRNA gene expression profiles of TNBC tumors from The Cancer Genome Atlas (TCGA)^26,27^, we apply the EDec^26^ computational cell type deconvolution method. Immune proportions of each tumor in the TCGA collection were determined, and the “hot” tumors were defined as those with immune proportions in the top quartile, while “cold” tumors were defined as those with immune infiltration in the bottom quartile.

**Fig. 1:**
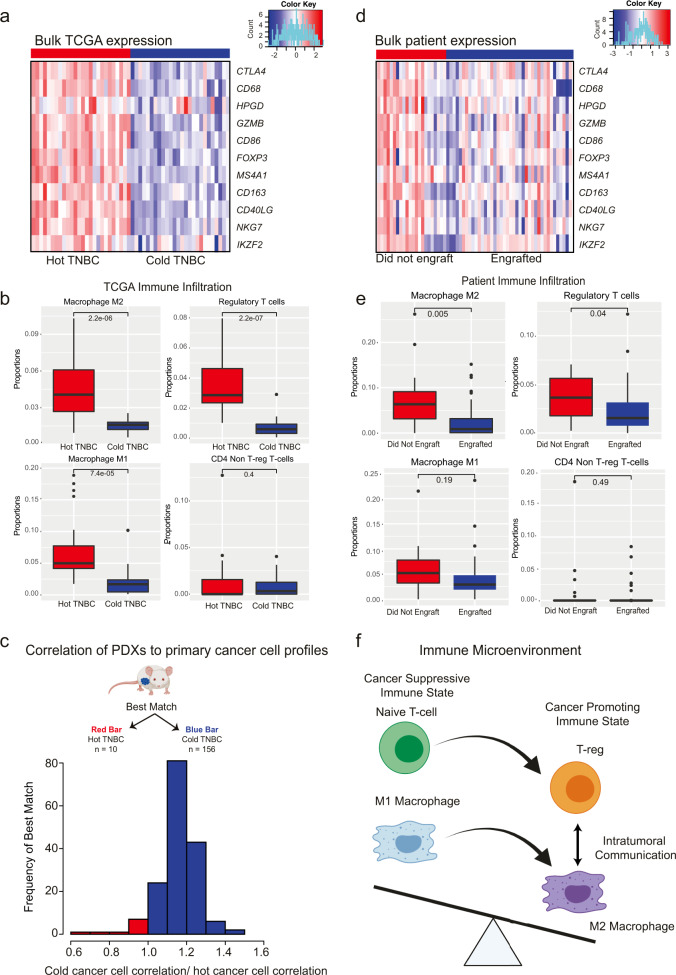
“Cold” TNBC show higher rates of engraftment in PDX models in contrast to immune enriched “Hot” TNBCs. **a** Bulk expression of immune genes in TCGA TNBC. Heatmap of the bulk expression of immune gene markers in hot and cold TNBC TCGA tumors (red bars and blue bars respectively). **b** Immune proportions in TCGA TNBC. Proportions of M2 macrophages (top left), T-regs (top right), M1 macrophages (bottom left), and non-T-reg CD4 T-cells (bottom right) in hot and cold TCGA TNBC tumors (red bars and blue bars respectively). T-tests were used to investigate the differences between hot and cold TNBC samples, and enter lines in the boxplots indicate the median. The bounds of the box indicate the first to third quartile values, while the bounds of the lower and upper whiskers indicate the smallest observation greater than or equal to the first quartile −1.5 times the inter-quartile range and the largest observation smaller or equal to the third quartile +1.5 times the inter-quartile range respectively. **c** Matching the PDX cancer cell profiles to the deconvoluted TCGA cancer cell profiles. 94% of the PDX models match the cold cancer cell profile. The histogram shows the frequency of the ratios of each model’s correlation the cold cancer cell profile vs the hot cancer cell profile. The bars in red (left) indicate that the hot cancer cell profile was the best match, while the bars in blue (right) indicate that the cold cancer cell profile was the best match. **d** Bulk expression of immune genes in patient tumors. Heatmap of bulk expression of immune cell markers in the HCI tumors that did not engraft in PDXs vs those that did (blue and red bars respectively). **e** Immune proportions in patient tumors. Proportions of M2 macrophages (top left), T-regs (top right), M1 macrophages (bottom left), and non-T-reg CD4 T-cells (bottom right) in hot and cold TCGA TNBC tumors (red bars and blue bars respectively). T-tests were used to investigate the differences between TNBCs that engrafted in murine models and those that did not. Center lines in the boxplots indicate the median, and the bounds of the box indicate the first to third quartile values. The bounds of the lower and upper whiskers indicate the smallest observation greater than or equal to the first quartile −1.5 times the inter-quartile range and the largest observation smaller or equal to the third quartile +1.5 times the inter-quartile range respectively. **f** Immune State. M2 macrophages release exosomes that include miR-29a-3p and miR-21-5p that reprogram T-cells to T-regs. Conversely, Tregs then release interleukins including IL-10, IL-4 and IL-13 that then reprogram M1 to M2 macrophages. The presence of T-regs and M2 macrophages leads to a cancer promoting tumor state.

As expected, Treg markers (*FOXP3, CTLA4*, *HPGD*, *IKZF2*), CD4 T-cell markers (*GZMB*, *NKG7*, *CD40LG*), M2 macrophage markers (*CD163*), and M1 macrophage markers (*CD68*, *CD86*), and B-cell marker (*MS4A1*) were all shown to be more highly expressed in the bulk expression profiles of hot TNBC group vs the cold TNBC group (Fig. 1a).

The cancer cell-specific gene expression profiles of hot TNBC and cold TNBC were then deconvoluted separately. The cold and hot groups also showed distinct epithelial cancer cell profiles, with 438 differentially expressed genes identified (at 5-fold difference between the cold and hot cancer cell profiles *p* < 0.05 thresholds, t-test). KEGG^28^ analysis showed that genes that were significantly upregulated in the hot vs cold cancer cell profiles were enriched for the cytokine-cytokine receptor interaction pathway (*p* = 0.02, hypergeometric test) (Table 1). Moreover, the GO^29^ analysis also showed an enrichment for pathways associated with immune activation (16/20 top pathways are associated with immune response) (Supplementary Table 1).

**Table 1:** KEGG analysis (hypergeometric test).

	Pathway	Genes Total	DE genes	*P* value
path: hsa04060	Cytokine-cytokine receptor interaction	295	3	0.02215201
path: hsa04390	Hippo signaling pathway	157	2	0.04182547
path: hsa04610	Complement and coagulation cascades	85	2	0.01331597
path: hsa05150	Staphylococcus aureus infection	96	2	0.01678076
path: hsa05417	Lipid and atherosclerosis	215	2	0.07321532

After characterizing the hot and cold primary TNBC cancer cell profiles, we then asked if the TNBC PDXs best matched the hot or cold cancer cell expression profiles. To address this question, we obtained RNA-seq data for 166 TNBC PDX models from the BCM and HCI collections, as well as other publicly available datasets (https://pdxportal.research.bcm.edu/pdxportal, https://pdmr.cancer.gov/, https://www.pdxfinder.org/, Table 2, Supplementary Table 2). The human cancer cell profiles of these PDXs were isolated by Xenome^25^, and the PDX cancer cell profiles were correlated to the deconvoluted cold and hot primary TCGA TNBC profiles. 94% (Fig. 1c*n* = 156 *p* < 0.001, binomial test) best matched cold cancer profile.

**Table 2:** PDX Summary statistics.

	TNBC PDX models	Unique patients
BCM	50	44
WHIM	9	6
HCI	26	25
UOM-BC	5	5
NKI	6	6
NCI PDMR	70	12
All Collections	166	98

To gain insights into the immunological mechanisms that may explain the engraftment bias, we then used quanTIseq^30^, an immune specific in silico deconvolution method, to perform an immune cell deconvolution of the hot and cold TNBC TCGA tumors. QuanTIseq does not estimate cell type-specific genes expression profiles but does allow for a more granular deconvolution of the tumor microenvironment. In hot TNBC TCGA tumors, there was a significantly higher proportion of M2 macrophage- (*p* < 0.001, t-test), T-reg- (*p* < 0.001 t-test), and M1 macrophage-related gene expression (*p* < 0.001, t-test) (Fig. 1b).

To explore the potential immunological influence on engraftment further, we then obtained RNA-seq data for 62 primary patient tumors that were implanted previously in PDX engraftment attempts (HCI collection, BCM collection, https://www.pdxfinder.org/, Table 3). Of these primary tumors 40 engrafted successfully, while 22 did not. As expected, the immune-related marker genes were found to be more highly expressed in the bulk expression profiles of the tumors that did not engraft (Fig. 1d). Quantiseq was used to deconvolute the immune fraction of these tumors. Tumors that failed to engraft in PDX models had a significantly higher proportion of total immune infiltration (*p* = 0.024, t-test). Both the proportions of T-regs (*p* = 0.04, t-test) and M2 macrophages (*p* = 0.005, t-test) were significantly higher in the samples that failed to engraft. T-regs and alternatively activated M2 macrophages both contribute to a cancer-promoting immune state, and have been previously implicated in promotion of tumor growth and proliferation. Additionally, intratumorally signaling between T-regs and M2 macrophages is associated with both tumor progression and a cancer-promoting immune state (Fig. 1f)^31–40^. Because samples that were enriched in T-reg and M2 macrophage signatures failed to engraft, we hypothesize that these samples had a cancer-promoting immune state that is not recapitulated in the immunodeficient PDX models.

**Table 3:** Matched patient summary statistics.

	Engrafted	Did not Engraft
BCM	30	15
NKI	5	0
HCI	5	7
All Collections	40	22

Taken together, our findings demonstrate that there is a bias towards the more successful engraftment of cold TNBC tumors as PDX models. Moreover, the immunologically hot tumors that do not engraft successfully as PDX models have an activated, growth-promoting immune cell state. Our result further suggests the intriguing possibility that an intact immune system of the PDX model may be essential for the growth of a majority of immunologically hot tumors. If confirmed by future research, this hypothesis would open the opportunity for exploring novel treatment options for hot TNBC by modulating the state of the host immune system. One major obstacle on that path of inquiry is that the “humanization” of mouse hosts by reconstitution of the human immune system has proven highly variable, as well as labor-intensive and expensive^41^. Thus, until immune system reconstitution in mice can be improved, our hypothesis of essentiality will be difficult to test.

Several other hypotheses could also explain the engraft bias towards cold TNBC tumors. Hot tumors may be targeted by the murine innate immune system, and thus be rejected despite the immunocompromised status of the host with respect to the adaptive immune system. Arguing against this hypothesis, our previous studies showed that the initial take rate (first passage in a host animal) for all breast cancer subtypes (~40%) is considerably higher than the proportion of tumors that can be established as stable PDX (~30% overall)^9,11^, suggesting that initial immune rejection does not completely explain lack of PDX engraftment. However, several studies have shown that superior engraftment of PDX occurs when mice lacking NK cells (e.g., NSG or SCID/Bg) are used, suggesting that at least this component of the innate immune system can limit engraftment. This was recently discussed in the context of breast cancer PDX models^11^. The specific roles of other innate immune cells (e.g., macrophages) in limiting PDX engraftment has not been determined.

Other systematic differences between the PDX models and “hot” TNBC tumors could also explain the engraftment bias. After engraftment, human stromal cells are replaced with murine stroma^42^, which was shown to be distinct from human stroma^43^. Cancer-Associated Fibroblasts (CAFs) promote angiogenesis and remodel the ECM. Additionally, CAFs in primary human tumors recruit T-regs and M2 macrophages and reprogram the immune system towards a cancer-promoting immune state^44^. Thus, differences in the murine and human stroma could also play a role in the failure of “hot” TNBC tumor engraftment.

Finally, it is also possible that engraftment may be a function of time to allow the hot donor tissue to adapt to the “cold” (lacking T-, B- and NK-cell) host environment, such that some tumors adapt quickly and grow, while others do not adapt in the timeframe of the useful lifespan of the immunocompromised host. In such a case, the lack of an immune system may or may not be the thing to which the tumor must adapt, as there are several other non-immune cell types present in the mammary gland (e.g., fibroblasts, adipocytes, vasculature) known to influence the growth of tumors. If true, serial transplantation of a donor tumor from the initial host to new hosts before the original host dies may allow growth of refractory tumor types such as immunologically hot TNBC, and perhaps also ER + and HER2 + breast cancers. Ultimately, better understanding of this modeling bias should help improve methods for engraftment, with subsequent increase in rigor, reproducibility, and translational potential of basic and pre-clinical cancer research involving mouse PDX models.

## Methods

### EDec deconvolution

TCGA TNBC RNA-Seq data was obtained from the UCSC Xena server (https://tcga.xenahubs.net) (Illumina HiSeq log2(x + 1) transformed RSEM normalized counts) and deconvoluted with EDec.

In Stage 1 of deconvolution, the immune proportion was determined for all TNBC tumors. This allowed for the identification of patients with low immune infiltration (bottom 25%) and high immune infiltration (top 25%). Stage 2 of EDec determines the cell type intrinsic expression profiles, and a separate Stage 2 deconvolution was performed for both the low immune component and the high immune component group. Differential gene expression was used to identify KEGG pathways that were differentially activated in the hot and cold TNBC cancer cell profiles.

### QuanTISeq deconvolution

quanTISeq was used to determine immune cell proportions for both TCGA tumors as well as the BCM and HCI tumors.

### Murine cancer cell profile matching

RNA-Seq data was obtained for the BCM and HCI PDX collection https://pdxportal.research.bcm.edu/ and the murine and human reads were separated with Xenome. The cancer reads were then correlated to the two cancer cell profiles obtained from the deconvolution of TGCA. RNA-seq for other PDX models was obtained from https://pdmr.cancer.gov/ and https://www.pdxfinder.org/.

### Statistical analysis

A binomial test was used to determine if there was an engraftment bias for hot vs cold patient tumors. The null hypothesis was that the PDXs represent the primary tumor population equally and 64% of the PDX models should match a cold basal profile^45^.

T-tests were used to determine *p*-values of the differences in the proportions of immune cells.

Informed consent was obtained for all human participants and all relevant ethical regulations were followed. The BCM institutional review board approved the study protocol.

## Supplementary information

Supplementary Information

## Data Availability

The data that supports the finding of this study is available from the following public databases: https://tcga.xenahubs.net, http://pdxportal.research.bcm.edu, https://pdmr.cancer.gov/, http://www.pdxnetwork.org, https://www.pdxfinder.org/, https://www.envigo.com/whim-pdx-models or upon reasonable request from M.T.L (GEO:GSE183187). Supplementary Table 3 provides further details on how to access each of the datasets utilized in this study.
