# Supplementary material for: Immunologically “cold” triple negative breast cancers engraft at a higher rate in patient derived xenografts
Source: NPJ Breast Cancer. 2022 Sep 10;8:104. doi: 10.1038/s41523-022-00476-0 (PMC9464188; doi:10.1038/s41523-022-00476-0)
Supplement: Supplementary file 1 — Supplementary Information [file 41523_2022_476_MOESM1_ESM.pdf]

Supplementary Table 1

Top 20 pathways in GO analysis (hypergeometric test)

	Pathway	Genes Total	DE genes	P-value
GO:0001775	cell activation	1482	8	0.00566265
GO:0002252	immune effector process	1299	9	0.00056144
GO:0002263	cell activation involved in immune response	720	8	4.89E-05
GO:0002274	myeloid leukocyte activation	662	7	0.00020949
GO:0002275	myeloid cell activation involved in immune response	549	7	6.55E-05
GO:0002283	neutrophil activation involved in immune response	490	7	3.19E-05
GO:0002366	leukocyte activation involved in immune response	716	8	4.70E-05
GO:0002376	immune system process	3287	13	0.00553053
GO:0002443	leukocyte mediated immunity	892	8	0.00021621
GO:0002444	myeloid leukocyte mediated immunity	555	7	7.02E-05
GO:0002446	neutrophil mediated immunity	501	7	3.68E-05
GO:0006810	transport	5224	19	0.00131266
GO:0006887	exocytosis	914	7	0.00142956
GO:0006955	immune response	2306	13	0.00019558

GO:0032940	secretion by cell	1436	9	0.0011524
GO:0036230	granulocyte activation	509	7	4.06E-05
GO:0042119	neutrophil activation	502	7	3.72E-05
GO:0043299	leukocyte degranulation	537	7	5.70E-05
GO:0043312	neutrophil degranulation	487	7	3.07E-05
GO:0045055	regulated exocytosis	797	7	0.00064152

Supplementary Table 2

PDX Characterization

PDX	Collection	ER IHC=	PR IHC	HER2 IHC
BCM-0002	BCM	Negative	Negative	Negative
BCM-0046	BCM	Negative	Negative	Negative
BCM-0049	BCM	Negative	Negative	Negative
BCM-0104	BCM	Negative	Negative	Negative
BCM-0113	BCM	Negative	Negative	Negative
BCM-0132	BCM	Negative	Negative	Negative
BCM-2147	BCM	Negative	Negative	Negative
BCM-2277	BCM	Negative	Negative	Negative
BCM-2665	BCM	Negative	Negative	Negative
BCM-3107	BCM	Negative	Negative	Negative
BCM-3204	BCM	Negative	Negative	Negative
BCM-3469	BCM	Negative	Negative	Negative
BCM-3561	BCM	Negative	Negative	Negative
BCM-3611	BCM	Negative	Negative	Negative
BCM-3807	BCM	Negative	Negative	Negative
BCM-3887	BCM	Negative	Negative	Negative
BCM-3904	BCM	Negative	Negative	Negative
BCM-3936	BCM	Negative	Negative	Negative
BCM-4013	BCM	Negative	Negative	Negative
BCM-4175	BCM	Negative	Negative	Negative
BCM-4195	BCM	Negative	Negative	Negative
BCM-4272	BCM	Negative	Negative	Negative
BCM-4400	BCM	Negative	Negative	Negative
BCM-4664	BCM	Negative	Negative	Negative
BCM-4849	BCM	Negative	Negative	Negative
BCM-4913	BCM	Negative	Negative	Negative
BCM-5156	BCM	Negative	Negative	Negative
BCM-5438	BCM	Negative	Negative	Negative
BCM-5471	BCM	Negative	Negative	Negative
BCM-5998	BCM	Negative	Negative	Negative
BCM-6257	BCM	Negative	Negative	Negative
BCM-7482	BCM	Negative	Negative	Negative
BCM-7563	BCM	Negative	Negative	Negative
BCM-7649	BCM	Negative	Negative	Negative

BCM-7821	BCM	Negative	Negative	Negative
BCM-8563	BCM	Negative	Negative	Negative
BCM-9161	BCM	Negative	Negative	Negative
BCM-15003	BCM	Negative	Negative	Negative
BCM-15006	BCM	Negative	Negative	Negative
BCM-15029	BCM	Negative	Negative	Negative
BCM-15046	BCM	Negative	Negative	Negative
BCM-15051	BCM	Negative	Negative	Negative
BCM-15115	BCM	Negative	Negative	Negative
BRA45	BCM	Negative	Negative	Negative
BRA74	BCM	Negative	Negative	Negative
GUM07	BCM	Negative	Negative	Negative
GUM13	BCM	Negative	Negative	Negative
GUM17	BCM	Negative	Negative	Negative
GUM30	BCM	Negative	Negative	Negative
MC1	BCM	Negative	Negative	Negative
HCI-001	HCI	Negative	Negative	Negative
HCI-002	HCI	Negative	Negative	Negative
HCI-003	HCI	Negative	Negative	Negative
HCI-004	HCI	Negative	Negative	Negative
HCI-005	HCI	Negative	Negative	Negative
HCI-006	HCI	Negative	Negative	Negative
HCI-007	HCI	Negative	Negative	Negative
HCI-008	HCI	Negative	Negative	Negative
HCI-009	HCI	Negative	Negative	Negative
HCI-010	HCI	Negative	Negative	Negative
HCI-011	HCI	Negative	Negative	Negative
HCI-012	HCI	Negative	Negative	Negative
HCI-013	HCI	Negative	Negative	Negative
HCI-015	HCI	Negative	Negative	Negative
HCI-016	HCI	Negative	Negative	Negative
HCI-017	HCI	Negative	Negative	Negative
HCI-018	HCI	Negative	Negative	Negative
HCI-019	HCI	Negative	Negative	Negative
HCI-023	HCI	Negative	Negative	Negative
HCI-024	HCI	Negative	Negative	Negative
HCI-025	HCI	Negative	Negative	Negative
HCI-026	HCI	Negative	Negative	Negative
HCI-027	HCI	Negative	Negative	Negative

HCI-028	HCI	Negative	Negative	Negative
HCI-030	HCI	Negative	Negative	Negative
HCI-041	HCI	Negative	Negative	Negative
WHIM2	WHIM	Negative	Negative	Negative
WHIM6	WHIM	Negative	Negative	Negative
WHIM12	WHIM	Negative	Negative	Negative
WHIM14	WHIM	Negative	Negative	Negative
WHIM30	WHIM	Negative	Negative	Negative
WHIM68	WHIM	Negative	Negative	Negative
WHIM69	WHIM	Negative	Negative	Negative
WHIM74	WHIM	Negative	Negative	Negative
WHIM75	WHIM	Negative	Negative	Negative
144163	UOM-BC	Negative	Negative	Negative
144201	UOM-BC	Negative	Negative	Negative
150467	NKI	Negative	Negative	Negative
144237	UOM-BC	Negative	Negative	Negative
144246	UOM-BC	Negative	Negative	Negative
150427	NKI	Negative	Negative	Negative
150452	NKI	Negative	Negative	Negative
150499	NKI	Negative	Negative	Negative
144184	UOM-BC	Negative	Negative	Negative
150483	NKI	Negative	Negative	Negative
150439	NKI	Negative	Negative	Negative
193832_AL-JGF	NCI PDMR Models	Negative	Negative	Negative
193832_AL-JGF_AL-WF0	NCI PDMR Models	Negative	Negative	Negative
193832_AL-JGF_AL-WF4	NCI PDMR Models	Negative	Negative	Negative
193832_AL-JGK	NCI PDMR Models	Negative	Negative	Negative
193832_AL-JGK_AL-W50	NCI PDMR Models	Negative	Negative	Negative
412248_QXC	NCI PDMR Models	Negative	Negative	Negative
412248_QXCXA7	NCI PDMR Models	Negative	Negative	Negative
412248_QXTTN3WN6	NCI PDMR Models	Negative	Negative	Negative
412248_QXTTN4	NCI PDMR Models	Negative	Negative	Negative
412248_QXTTN7	NCI PDMR Models	Negative	Negative	Negative
412248_QXUVX9	NCI PDMR Models	Negative	Negative	Negative
414838_AL-Y5CXC8YA9	NCI PDMR Models	Negative	Negative	Negative
414838_AL-Y5C_AL-XC6	NCI PDMR Models	Negative	Negative	Negative
414838_AL-Y5E	NCI PDMR Models	Negative	Negative	Negative
414838_AL-Y5EWY7	NCI PDMR Models	Negative	Negative	Negative
414838_AL-Y5E_AL-WY3YK7	NCI PDMR Models	Negative	Negative	Negative

414838_AL-Y5F_AL-XW4	NCI PDMR Models	Negative	Negative	Negative
415267_AK3	NCI PDMR Models	Negative	Negative	Negative
415267_AK3JG4	NCI PDMR Models	Negative	Negative	Negative
415267_AK3JG5K66	NCI PDMR Models	Negative	Negative	Negative
415267_AK3JG5K68C67	NCI PDMR Models	Negative	Negative	Negative
415267_AK3JJ0C83	NCI PDMR Models	Negative	Negative	Negative
415267_AK6	NCI PDMR Models	Negative	Negative	Negative
447297_AL-WYC	NCI PDMR Models	Negative	Negative	Negative
447297_AL-WYC_AL-GV6	NCI PDMR Models	Negative	Negative	Negative
447297_AL-WYC_AL-GV7	NCI PDMR Models	Negative	Negative	Negative
447297_AL-WYC_AL-GV7_AL-A32_AL-N37_AL-T78	NCI PDMR Models	Negative	Negative	Negative
447297_AL-WYC_AL-GV7_AL-A32_AL-N45	NCI PDMR Models	Negative	Negative	Negative
556579_UWTJT3	NCI PDMR Models	Negative	Negative	Negative
556579_UWU	NCI PDMR Models	Negative	Negative	Negative
556579_UWUJJ0KX2	NCI PDMR Models	Negative	Negative	Negative
556579_UWUJJ1_RG-PV8	NCI PDMR Models	Negative	Negative	Negative
556579_UWUJJ3	NCI PDMR Models	Negative	Negative	Negative
556579_UWVJT7	NCI PDMR Models	Negative	Negative	Negative
562452_AL-KV5P13	NCI PDMR Models	Negative	Negative	Negative
562452_AL-KV7_AL-C01P33	NCI PDMR Models	Negative	Negative	Negative
562452_AL-KV7_AL-C02	NCI PDMR Models	Negative	Negative	Negative
562452_AL-KV8	NCI PDMR Models	Negative	Negative	Negative
562452_AL-KV9	NCI PDMR Models	Negative	Negative	Negative
562452_AL-KV9_AL-J89	NCI PDMR Models	Negative	Negative	Negative
731979_AL-VAY_AL-W08	NCI PDMR Models	Negative	Negative	Negative
731979_AL-VAY_AL-W10	NCI PDMR Models	Negative	Negative	Negative
731979_AL-VAY_AL-W13_AL-P28	NCI PDMR Models	Negative	Negative	Negative
731979_AL-VAY_AL-W13_AL-P28_AL-U19	NCI PDMR Models	Negative	Negative	Negative
731979_AL-VAY_AL-W13_AL-P30	NCI PDMR Models	Negative	Negative	Negative
731979_AL-VJW	NCI PDMR Models	Negative	Negative	Negative
868763_AL-N2P	NCI PDMR Models	Negative	Negative	Negative
868763_AL-N2P_AL-Y21	NCI PDMR Models	Negative	Negative	Negative
868763_AL-N3P	NCI PDMR Models	Negative	Negative	Negative
868763_AL-N3P_AL-X21	NCI PDMR Models	Negative	Negative	Negative
868763_AL-NTT_AL-X86	NCI PDMR Models	Negative	Negative	Negative

868763_AL-NTT_AL-X92	NCI PDMR Models	Negative	Negative	Negative
885512_AL-KAE_AL-Q12	NCI PDMR Models	Negative	Negative	Negative
885512_AL-KAF	NCI PDMR Models	Negative	Negative	Negative
885512_AL-KAF_AL-Q24	NCI PDMR Models	Negative	Negative	Negative
885512_AL-KAG_AL-Q33	NCI PDMR Models	Negative	Negative	Negative
885512_AL-KAG_AL-Q34_AL-T39	NCI PDMR Models	Negative	Negative	Negative
885512_AL-KAW_AL-Q88	NCI PDMR Models	Negative	Negative	Negative
974727_OT-PPJ	NCI PDMR Models	Negative	Negative	Negative
974727_OT-PPJ_OT-UH0YJ3	NCI PDMR Models	Negative	Negative	Negative
974727_OT-PPJ_OT-UH3XX0	NCI PDMR Models	Negative	Negative	Negative
974727_OT-PPJ_OT-UH3XX3CK1	NCI PDMR Models	Negative	Negative	Negative
974727_OT-PPJ_OT-UH3XX3CK4F16	NCI PDMR Models	Negative	Negative	Negative
974727_OT-PPJ_OT-UH5	NCI PDMR Models	Negative	Negative	Negative
994819_OT-FWCP10	NCI PDMR Models	Negative	Negative	Negative
994819_OT-FWF	NCI PDMR Models	Negative	Negative	Negative
994819_OT-FWFP18_RG-N13	NCI PDMR Models	Negative	Negative	Negative
994819_OT-FWFP18_RG-N15	NCI PDMR Models	Negative	Negative	Negative
994819_OT-FWFP18_RG-N15K11	NCI PDMR Models	Negative	Negative	Negative
994819_OT-FWFP19	NCI PDMR Models	Negative	Negative	Negative

Supplementary Table 3

Datasets Utilized

Collection	Tumor Type	Number of Samples	Resource Location
TCGA TNBC	Primary Human	100 TNBC	https://tcga.xenahubs.net
BCM (Patient)	Primary Human	45	https://pdxportal.research.bcm.edu<br>mtlewis@bcm.edu
NKI (Patient)	Primary Human	5	https://www.pdxfinder.org/
HCI (Patient)	Primary Human	12	alana.welm@hci.utah.edu
BCM and HCI	PDX	76 TNBC PDXs	https://pdxportal.research.bcm.edu
NCI PDMR	PDX	70 TNBC PDX	https://pdmr.cancer.gov/database/default.htm
NKI	PDX	6 TNBC PDX	https://www.pdxfinder.org/
UOM-BC	PDX	5 TNBC PDX	https://www.pdxfinder.org/
WHIM	PDX	9 TNBC PDX	https://www.envigo.com/whim-pdx-models

`
